# Molecular characterization of *Histoplasma capsulatum *isolated from an outbreak in treasure hunters *Histoplasma capsulatum *in treasure hunters

**DOI:** 10.1186/1471-2334-10-264

**Published:** 2010-09-08

**Authors:** Bertha Muñoz, María Á Martínez, Gabriel Palma, Amado Ramírez, María G Frías, María R Reyes, María L Taylor, Anjarath L Higuera, Alexander Corcho, María E Manjarrez

**Affiliations:** 1 Laboratorio de Micología Médica, Depto. de Investigación en Virología, Instituto Nacional de Enfermedades Respiratorias (INER), Calzada de Tlalpan 4502, Sección XVI, Tlalpan,14080 México, D.F., México; 2 Laboratorio de Micología Médica, Depto. de Microbiología, Escuela Nacional de Ciencias Biológicas (ENCB), Instituto Politécnico Nacional (IPN), México, D.F., México; 3 Laboratorio de Biología Molecular de Hongos, Depto. de Microbiología y Parasitología, Facultad de Medicina, Universidad Nacional Autónoma de México (UNAM), Mexico City, Mexico; 4 Laboratorio de Inmunología de Hongos, Depto. de Microbiología y Parasitología, Facultad de Medicina, (UNAM), Mexico City, Mexico; 5 Departamento de Investigación en Epidemiología Clínica, Nacional de Enfermedades Respiratorias (INER), Calzada de Tlalpan 4502, Sección XVI, Tlalpan,14080 México, D.F., México

## Abstract

**Background:**

In Mexico, primary pulmonary histoplasmosis is the most relevant clinical form of the disease. The geographical distribution of specific strains of *Histoplasma capsulatum *circulating in Mexico has not been fully established. Outbreaks must be reported in order to have current, updated information on this disease, identifying new endemic areas, manner of exposure to the fungi, and molecular characterization of the causative agents. We report a recent outbreak of histoplasmosis in treasure hunters and the molecular characterization of two isolates obtained from these patients.

**Methods:**

Six patients admitted to the National Institute of Respiratory Diseases (INER) in Mexico City presented severe respiratory symptoms suggestive of histoplasmosis. They acquired the infection in the Veracruz (VZ) endemic zone. Diagnosis was made by X-ray and Computed tomography (CT), liver function, immunological techniques, and culture. Identification of *H. capsulatum *isolates was confirmed by using Polymerase chain reaction (PCR) was conducted with a probe from the M antigen, and the isolates were characterized by means of Random amplification of polymorphic DNA (RAPD)-PCR employed the 1253 oligonucleotide and a mixture of oligonucleotides 1281 and 1283. These were compared to eight reference strain isolates from neighboring areas.

**Results:**

X-ray and CT revealed disseminated micronodular images throughout lung parenchyma, as well as bilateral retrocaval, prevascular, subcarinal, and hilar adenopathies, hepatosplenomegaly, and altered liver function tests. Five of the six patients developed disseminated histoplasmosis. Two *H. capsulatum *strains were isolated. The same band profile was detected in both strains, indicating that both isolates corresponded to the sole *H. capsulatum *strain. Molecular characterization of the isolates was similar in 100% with the EH-53 Hidalgo human (HG) strain (reference strain integrated into the LAm A clade described for Latin America).

**Conclusions:**

The two isolates appeared to possess the same polymorphic pattern; they are indistinguishable from each other and from EH-53. It is important to remain updated on recent outbreaks of histoplasmosis, the manner of exposure to the fungi, as well as the molecular characterization of the isolates. The severity of cases indicates that this strain is highly virulent and that it is probably prevalent in Hidalgo and Veracruz states.

## Background

*Histoplasma capsulatum *develops in bird excrement or bat guano in closed areas (caves, mines, tunnels) or in open spaces (parks, orchards, and abandoned homes, among others). The organic material found in these waste materials under humid conditions and optimal temperatures establishes the ecological niche for the mycelial phase of development, and microconidium constitutes the infectious form of the fungus [1-3]. There have been three distinct genotypes and varieties of *H. capsulatum*, and two have infected human beings: *H. capsulatum *var. *capsulatum*, and *H. capsulatum *var. *duboisii*; in addition, one has infected equines (*H. capsulatum *var. *farciminosum*). These exhibited different clinical manifestations and geographical distributions [4-6]. However, a phylogenetic study by Kasuga and colleagues [7,8] was conducted in an attempt to resolve the relationships among the major classes and three varieties of *H. capsulatum*. At least eight clades were identified: (i) North American class 1 clade; (ii) North American class 2 clade; (iii) Latin American group A clade; (iv) Latin American group B clade; (v) Australian clade; (vi) Netherlands (Indonesian?) clade; (vii) Eurasian clade, and (viii) African clade. Seven of the eight clades represented genetically isolated groups that may be recognized as phylogenetic species.

This fungus is the etiological agent of systemic mycosis histoplasmosis. The mycosis is an endemic disease of tropical, subtropical, and temperate areas throughout the world. Histoplasmosis has been described as present in all of the 32 states of the Mexican Republic, with highly variable prevalence [3,9-19]. Veracruz is one of the federal entities with the greatest number of cases reported [18]. In Mexico, histoplasmosis is considered an occupational disease that especially affects miners, farmers, guano collectors, cockfight breeders, geologists, speleologists, anthropologists, and biologists. Primary pulmonary histoplasmosis (PPH) is the most relevant clinical form of the disease with the highest mortality rate worldwide [10,11,18,20]. The clinical manifestations of this mycosis may range widely, from being clinically benign to severe, even fatal, depending upon the amount of propagules inhaled, the immunological state of the infected host, and the strain's virulence [3]. It is important to report outbreaks of histoplasmosis in order to know the current status of this disease, identify new endemic areas, and for molecular characterization of the causative agents. This study reports on an outbreak of histoplasmosis in treasure hunters in the endemic zone of Veracruz and the molecular characterization of two isolates from these patients.

## Methods

### Case studies

**Figure 1:**
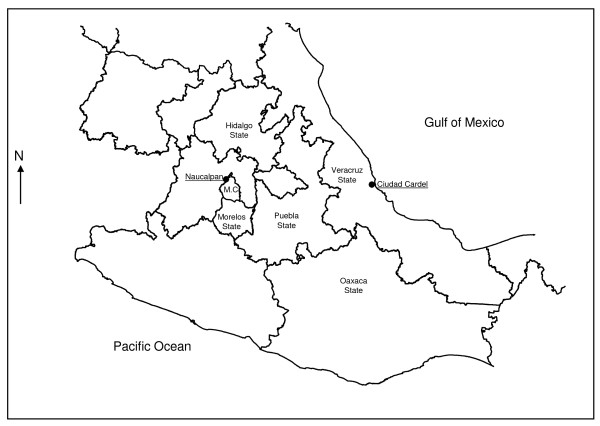
**Ciudad Cardel, La Antigua Municipality, Veracruz, Mexico**. The figure shows, the Veracruz-neighboring states, endemic areas from which isolates and type strains and, Mexico City (MC) and Naucalpan (patient's place of residence).

Six patients (two children and four adults), one with type 2 diabetes mellitus, were admitted to the Instituto Nacional de Enfermedades Respiratorias (INER) in Mexico City in August 2007 with severe histoplasmosis-associated respiratory symptoms. On questioning, patients referred having family ties among themselves and that they were residents of Naucalpan, near Mexico City. They had traveled to the town of Tamarindos, Ciudad Cardel, Veracruz (a histoplasmosis endemic zone) (Figure 1) in a search for treasure. They had slept for 3 consecutive days in a house and also mentioned that they excavated for 2 days with constant exposure to dust, migratory bird excreta, as well as bat guano in surrounding areas.

Informed consent was obtained from patients or their parents or guardians, and the Human Experimentation Guidelines of the Institution were followed according to clinical research conduct.

### Isolation and identification of H. capsulatum and detection of anti-H. capsulatum antibodies

The specimens analyzed included bronchial washings, sputum, and blood. All samples were sent to the laboratory for immediate processing. Direct examination from specimens with 15% potassium hydroxide was performed, as well as cytological tests with methenamine silver (Grocott) and Periodic acid-Schiff (PAS) staining, in Sabouraud dextrose-agar (SDA) cultures, and SDA with antibiotics (chloramphenicol, 50 mg/L, and cycloheximide, 500 mg/L). Isolates were identified on the basis of colonial and microscopic morphology. Serological tests for anti-*H. capsulatum *antibodies included Capillary tube precipitation (CTP) [21], gel Immunodiffusion (ID) [22-24], and the indirect Enzyme linked immunosorbent assay (ELISA) method according to Voller et al. [25]. Immune cell response was assessed by means of skin testing; this method evaluates the reactivity of the patients to histoplasmin. Changes observed in lungs, liver, and spleen were assessed utilizing X-rays and CT scans.

### Molecular identification and characterization of isolates

Molecular typing of the two isolates was performed employing Polymerase chain reaction (PCR). *H. capsulatum *reference strains were used from Mexican patients with disseminated histoplasmosis: EH-53 from Hidalgo (HG) and EH-317 human from Morelos (MS), and the following isolates from infected bats: EH-397 *Pterotus davyi*, and EH-398 *Leptonycteris curasoae *from Oaxaca (OC); EH-406 *Leptonycteris novalis *and EH-408 *Leptonycteris novalis *from Puebla (PL), and EH-437 *Desmodus rotundus *and EH-449 *Leptonycteris novalis *from Morelos (MS), all of these areas located near the state of Veracruz. All of these strains are labeled as follows: "*Histoplasma capsulatum *Strain Collection of the Fungal Immunology Laboratory of the Department of Microbiology-Parasitology, Faculty of Medicine, National Autonomous University of Mexico (UNAM)", which is registered in the World Data Centre for Microorganisms (WDCM) database under the acronym LIH-UNAM WDCM817. Information on strains is available at the website [26].

### DNA extraction

The biomass from each isolate was obtained by filtering the mycelial growth developed in GYE medium (glucose 2%, yeast extract 1%), and DNA was obtained as described by Reyes-Montes et al. [27].

### PCR

The reaction was carried out utilizing the oligonucleotides reported by Guedes et al. [28] with the following modifications: in a 25-μL final reaction volume, we used 30 ng of genomic DNA, 2.0 mM MgCl_2_, 200 μM dNTPs (Applied Biosystems, Inc., Foster City, CA, USA), 1.0 U Taq polymerase (Applied Biosystems), and 50 pmol/μL of each oligonucleotide. The amplification program comprised one 3-min cycle at 95°C followed by 35 1-min cycles at 95°C, 1 min at 55°C, 1 min at 72°C, and a final 5-min cycle at 72°C to ensure full extension of all amplified products. The amplified products were analyzed in 1.5% agarose gel. Electrophoresis was conducted at 100 V in TBE 0.5 × buffer. The molecular size standard employed was 100-bp DNA Ladder (Invitrogen, Carlsbad, CA, USA).

### RAPD-PCR

Random amplification polymerase DNA (RAPD) *H. capsulatum *DNAs were amplified employing the oligonucleotides 1253 (5'-GTTTCCGCCC-3') and a double initiator, 1281 (5' AACGCGCAAC-3') and 1283 (5'-GCGATCCCCA-3') (Operon Technologies, Inc., Alameda, CA, USA) according to Kersulyte et al. [29] and Woods et al. [30]. The reaction was performed according to Reyes-Montes et al. [27] and Taylor et al. [31]. Amplicons were observed by staining the gels with ethidium bromide (10 μg/mL). A 100-bp DNA Ladder (Invitrogen) was utilized as standard molecular size. Images of the gels were registered in a Synoptics Photodocumenter (Syngene, Cambridge, MA, USA). A dendrogram was constructed from the molecular profiles obtained to correlate the percentage of similarity among strains. The dendrogram was generated using the Unweighted pair group method with arithmetic mean (UPGMA), and Jaccard co-efficient was estimated from a band presence-and-absence matrix.

## Results

### Case Studies

The infection site is located in the town of Tamarindos, Ciudad Cardel, Veracruz state, in a histoplasmosis endemic zone (Figure 1). Average time for symptoms to appear was 7 to 20 days. Main clinical manifestations included fever, bouts of dry cough, chest pain, dyspnea with medium effort, shivering, diaphoresis, myalgias, arthralgias, headaches, weight loss, nausea, vomiting (these latter two symptoms are rare in this infection) [32,33], and an overall deficient health state. The clinical forms of the disease and organs affected are listed in Table 1. Disseminated histoplasmosis was considered when patients had hepatosplenomegaly and elevated liver enzymes, especially alkaline phosphatase. In all cases, chest x-rays and high-resolution CT scan showed micronodular images in both lungs. Moreover, in patients with disseminated histoplasmosis, the chest CT scan divulged bilateral retrocaval, prevascular, subcarinal, and hiliar adenopathies, and hepato- and splenomegaly.

**Table 1:** Demographic and clinical data patients

Case	Gender	Age (years)	Occupation	Incubation period (days)	Time disease evolution (days)	Clinical form	Organs affected
1	M	49	Taxi driver	7	25	PPH	Lungs
2	M	27	Gas-station attendant	7	25	Disseminated histoplasmosis	Lungs, liver, and spleen
3	M	46	Shaman	20	12	Disseminated histoplasmosis	Lungs, liver
4	F	36	Home maker	8	26	Disseminated histoplasmosis	Lungs, liver, and spleen
5	F	11	Student	10	25	Disseminated histoplasmosis	Lungs, liver, and spleen
6	F	8	Student	10	25	Disseminated histoplasmosis	Lungs, liver, and spleen

M, Male; F, Female; PPH, Primary pulmonary histoplasmosis.

#### Isolation and Identification of H. capsulatum and detection of anti-H. capsulatum antibodies

Cytology of the specimens exhibited the yeast phase of the fungus. Only two isolates were obtained from different patients: LFD, clinical isolate (bronchial lavage), and EVP clinical isolate (sputum), which were totally identified based on inclusion criteria for the *H. capsulatum *(type B colonies) species and its characteristic macro- and micromorphology. Serologic results were the following: PT, 1:32 to 1:256; ELISA, >1:320; H and M bands were observed in ID, and Intradermoreaction (IDR) to histoplasmin (>5-mm induration) was noted in all cases.

#### Molecular Characterization of the Isolates

**Figure 2:**
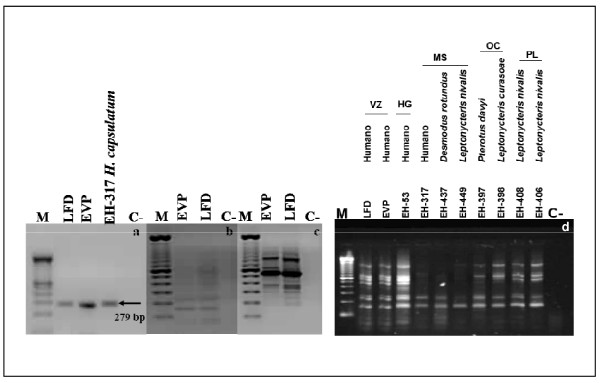
**Identification and characterization of *Histoplasma capsulatum *isolates from Veracruz (VZ)**. (a) Identification of *H. capsulatum *isolates using Polymerase chain reaction (PCR) with the probe designed from the M antigen; (b and c) characterization by Random amplification of polymorphic DNA-PCR (RAPD-PCR) of *H. capsulatum *isolates with 1253 (b) and the mixture of 1281 and 1283 oligonucleotides (c); M 100 bp DNA Ladder; LFD, EVP, and EH-317 *H. capsulatum*; C (-) reagent control. (d) RAPD-PCR DNA patterns obtained from *H. capsulatum *isolates LFD and EVP compared with isolated 8 reference strains from humans or bats from different geographical regions in Mexico.

**Figure 3:**
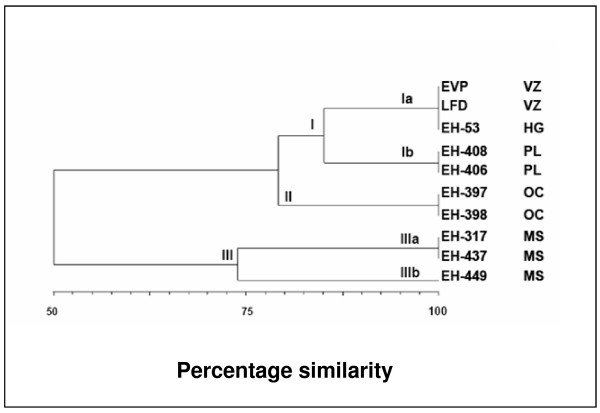
**Dendrogram constructed from the molecular profiles**. Genetic similarity among *Histoplasma capsulatum *isolates from Veracruz (VZ) with isolates from other neighboring endemic zones, in Mexico.

Molecular identification of the isolates was confirmed by PCR on amplifying a specific 279 base-pair (bp) band corresponding to the codifying region for the *H. capsulatum-*immunodominant M-antigen gene [28] (Figure 2a). Molecular characterization of the two clinical isolates of *H. capsulatum *from VZ was conducted oligonucleotides 1253 (figure 2b) and 1281 and 1283 (figures 2c and 2d) RAPD-PCR assays. The figure 2d shows the molecular profiles of these two isolates, compared with those of the eight *H.-capsulatum *reference strains. The bands obtained were located in the 800 to 200-bp range (Figure 2d). Relatedness among isolates through the polymorphic DNA patterns was analyzed by using the UPGMA program to elaborate a dendrogram (Figure 3) and three groups, according to RAPD-patterns, were identified. Group I is made up of two subgroups: subgroup 1a, which includes VZ (LFD [bronchial lavage] and EVP [sputum]) isolates, and the EH-53 (HG) reference strain, which exhibited 100% similarity, while subgroup Ib comprised bat isolates from PL. Both subgroups are related at 85% similarity. Group II comprises of EH-397 and -398 bat isolates from (OC) with 100% similarity between them. This group is related to group I with 82% similarity. Group III is made up of two subgroups: subgroup IIIa is formed by EH-317 and EH-437 bat isolates from MS, with a 100% similarity ratio, and subgroup IIIb consists of isolates from the State of Morelos (EH-449), which were 73% related to subgroup IIIa strains Finally, group III is related to the previous groups with 52% similarity.

## Discussion

In Mexico, the first probable outbreak of histoplasmosis was described and recorded in a Public Health Acts ledger from the state of Nuevo León at the end of the 19^th ^century (ca. 1885) when certain clinical scenarios were described that led to the suspicion that there was an epidemic of this mycosis in mine workers from areas containing a large amount of bat guano. In 1943, Martínez-Báez reported the first proven case of histoplasmosis in Mexico, while the first officially described outbreak was observed in subjects who had entered into "El Refugio", an abandoned mine located in Lampazos, Nuevo León; both data were referred by Aguirre-Pequeño [34].

From studies by Aguirre-Pequeño [34] and González-Ochoa [9-13], histoplasmosis in Mexico is considered an occupational disease because a relationship has been established between severe Primary pulmonary histoplasmosis (PPH) in miners exposed to high concentrations of *H.-capsulatum *propagules from bat guano. Histoplasmosis outbreaks have reported with a death rate as high as 100% in affected individuals [18-20]. Despite that the number of histoplasmosis outbreaks continues to rise, there are no official records of the majority of these. The most recent outbreak reported occurred in Spring-Break student tourists in Acapulco between March and May 2001 [35]. During this outbreak, *H. capsulatum *was isolated from compost employed as fertilizers in ornamental plants at the hotel where the students stayed [17].

The results of our study show that the patients had acquired histoplasmosis in the town of Tamarindos, Ciudad Cardel, Veracruz (Figure 1). Patients' signs and symptoms were clearly associated with chest x-ray images and chest CT scans. The etiological agent was clearly identified utilizing macro- and micromorphology and assessment of anti-Histoplasma antibodies, and IDR, and its identification was confirmed by PCR (Figure 2a). In addition, using RAPD-PCR, the two isolates were observed to possess the same polymorphic pattern based on their genetic material, indicating that the patients were infected with the same *H. capsulatum *strain prevalent at this site (Figures 2b and 2c).

In this study, five of the six patients developed disseminated histoplasmosis. The severity of the infection in these patients can be attributed to factors such as i) extreme physical effort, ii) inhalation of a large number of infectious propagules (when digging to find a treasure, the moving of the bat and bird excreta found at the infection site), iii) long exposure time (patients remained for 3 full days excavating and sleeping at the same site), iv) low socioeconomic status of the workers with severe nutritional and immunological deficiencies, and v) a probable high virulence of the strains present at the infection site. The EH-53 reference strain is highly virulent, proceeding from HG, Mexico, and isolated from a patient with fatally disseminated histoplasmosis. Although the molecular characterization of isolates was not carried out in full, 100% similarity (observed in the dendrogram) between the two VZ isolates and the EH-53 strain (Figure 3), and the severity of the patients' clinical problems, could suggest the presence of the strain in this geographic area. Several authors have reported a high virulence rate of *H. capsulatum *strains in Mexico [10,11,18,20,36].

Molecular typification of the two isolates from this outbreak was compared with the polymorphic DNA patterns of strains collected from VZ-neighboring areas. EVP and LFD isolates presented 100% similarity to the EH-53 HG strain (Figure 3), which was isolated in 1977; therefore, the two isolates are considered as probably the same strain. The EH-53 strain is included in the Lam A clade (forming a monophyletic group for Latin America) according to Kasanuga et al. [8]. Due to that these isolates were not characterized by four genes as utilized by Kasuga because we did not possess the appropriate primers, it was not possible to perform a definitive classification.

The Ciudad Cardel, La Antigua, Municipality, Veracruz (Figure 1), endemic zone where patients acquired the infection is located in the central coastal zone of the state of Veracruz at coordinates 19°22' latitude North and 96° 22' longitude West, and at an altitude of 20 m above sea level. Its climate is warm with an average temperature of 25°C. Average annual rainfall is 1,500 mm. In this municipality, fauna is mainly comprised of rabbits, foxes, coyotes, armadillos, raccoons, and bats; reptiles such as rattlesnakes, migratory birds, and other birds are often found to be *H. capsulatum *reservoirs. The house where the patients slept was surrounded by trees in which bats and migratory birds roosted overnight. Maintenance of this strain in the environment, in the neighboring areas of both HG and VZ, could probably be explained by the very important role that bat guano plays in spreading *H. capsulatum*, as reported by Taylor et al. [16], in which the authors mention that *H. capsulatum *propagules can be dispersed at different distances. In general, it appears that bats are the ideal candidates for spreading *H. capsulatum *in both short, as well as in long, distances. The cave-colonizing behavior of bats, their ability to fly, and their habit of remaining in the same caves for long periods are important factors that explain the dynamics behind the dispersion of the fungus in nature. For example, infected bats can act as dispersers of the parasite and incorporate the fungus into new, favorable environments, possibly through cadavers [15]. Additionally, the wide diversity of the fauna present, which is characteristic of the infectious site, also contributes importantly to maintenance of *H. capsulatum *in the environment.

The therapeutic scheme administered to three patients was amphotericin B deoxycholate (0.7 mg/kg/day every 7 days), and methylprednisone (1 mg/kg/day every 7 days), prior to administering amphotericin B, to avoid collateral effects; afterward, itraconazole 600 mg/day was administered for 3 days, and finally, itraconazole 400 mg/day was given every 12 weeks), while the remaining three patients were only administered itraconazole (200 mg/day every 12 weeks). Symptoms and lesions in all patients resolved. The symptomatology of the febrile respiratory disease is not specific for pulmonary histoplasmosis, and on occasion, a mistaken diagnosis is reached, with subsequent administration of the inappropriate treatment.

## Conclusions

The two isolates were observed as possessing the same polymorphic pattern, indicating that the patients were infected with the same *H. capsulatum *strain prevalent at this site. It is important to remain updated on recent outbreaks of histoplasmosis and the manner of exposure to the fungi, as well as to emphasize molecular characterization of isolates located in this endemic zone. The severity of cases indicates that these strains are highly virulent, and it is probable that these isolates are prevalent in Hidalgo and Veracruz states. People residing in or visiting this endemic area should be aware of the health risks of histoplasmosis infection to which they might be subjected, to as well as health care personnel for them to exercise the necessary preventive measures.

## Competing interests

The authors declare that they have no competing interests.

## Authors' contributions

BM Participated in study design and coordination and helped to draft the manuscript. MAM Participated in sequence alignment and drafted the manuscript. GP Performed the serologic tests and obtained the samples. AR Participated in the clinical studies and patient data case studies. MGF Conducted the molecular studies. MRR Performed and interpreted the molecular studies. MLT Assisted in characterization of isolates with reference strains. ALH Participated in clinical and case studies and performed data analysis. AC Obtained the samples and performed the data analysis. MEM Conceived of the study, participated in its design, and drafted the manuscript.

All authors read and approved the final manuscript.

## Pre-publication history

The pre-publication history for this paper can be accessed here:

http://www.biomedcentral.com/1471-2334/10/264/prepub
